# Proposed Novel Heart Failure Biomarkers and Their Association with Length of Hospital Stay and Mortality: A Retrospective Observational Pilot Study

**DOI:** 10.3390/diagnostics15050589

**Published:** 2025-02-28

**Authors:** Liviu Cristescu, Dragos-Gabriel Iancu, Marius-Stefan Marusteri, Ioan Tilea, Andreea Varga

**Affiliations:** 1 Doctoral School, George Emil Palade University of Medicine, Pharmacy, Science and Technology of Targu Mures, 540142 Targu Mures, Romania; liviu.cristescu@umfst.ro; 2 Faculty of Medicine, George Emil Palade University of Medicine, Pharmacy, Science and Technology of Targu Mures, 540142 Targu Mures, Romania; marius.marusteri@umfst.ro (M.-S.M.); ioan.tilea@umfst.ro (I.T.); 3 Faculty of Medicine in English, George Emil Palade University of Medicine, Pharmacy, Science and Technology of Targu Mures, 540142 Targu Mures, Romania; andreea.varga@umfst.ro

**Keywords:** chronic heart failure, biomarkers, NT-proBNP, NTAR, RGR, RFR, length of hospital stay, in-hospital mortality, 6-month all-cause mortality

## Abstract

**Background/Objectives:** Chronic heart failure (CHF) remains a significant global health burden, with high morbidity, prolonged hospitalizations, and increased mortality. Traditional biomarkers such as NT-proBNP provide prognostic value; however, novel biomarker ratios may enhance risk stratification. This study evaluated the predictive utility of the NT-proBNP-to-albumin ratio (NTAR), red cell distribution width-to-eGFR ratio (RGR), and red cell distribution width-to-fibrinogen ratio (RFR) for hospital length of stay (LOS), extended hospitalization (ELOS), in-hospital mortality, and 6-month all-cause mortality. **Methods:** A retrospective observational pilot study was conducted on 382 CHF admissions (2022–2024) with comprehensive laboratory assessment. Biomarker performance was assessed through uni- and multivariate logistic regression, receiver operating characteristic curve, and Cox proportional hazards stepwise methods of analyses for refining predictive models. **Results:** NTAR and RGR emerged as significant predictors of hospitalization outcomes. NTAR demonstrated a moderate correlation with prolonged LOS (r = 0.45, *p* < 0.001) and was an independent predictor of ELOS (AUC = 0.697, OR = 2.438, *p* < 0.001), outperforming NT-proBNP. Additionally, NTAR significantly predicted in-hospital mortality (AUC = 0.768, OR = 4.461, *p* < 0.001) and 6-month all-cause mortality (AUC = 0.766, OR = 4.185, *p* < 0.001). RGR was the strongest predictor of in-hospital mortality (AUC = 0.785, HR = 2.18, *p* = 0.005), highlighting its role in renal dysfunction and erythropoietic alterations in CHF. The RFR observed prognostic value was minimal. **Conclusions:** In our study, NTAR and RGR offered valuable prognostic value underscoring the interplay of cardiac stress, nutritional status, and renal function in CHF prognosis. Further multicenter validation is warranted for these biomarkers.

## 1. Introduction

Heart failure (HF) is a multifaced clinical syndrome involving increased mortality and morbidity [1]. In 2019, the Heart Failure Association (HFA) Atlas survey conducted across 13 European countries estimated the prevalence of HF in Europe at approximately 17 cases for every 1000 individuals. Due to limited data availability and considerable high heterogeneity regarding mortality, accurate data are not available across Europe as assessed by the HFA of the ESC [2]. In the 2022 European Society of Cardiology (ESC)—HFA position-paper, Rosano et al. synthesized data from multiple studies, revealing over 63 million individuals suffering from HF globally, with a mere 50% survival rate beyond 5 years [3].

According to data from the ESC e-Atlas, the mean length of hospital stay (LOS) for HF patients in Europe in 2019 was 8.5 days, whereas HF-related hospitalizations in Romania had an average LOS of 7.3 days [4]. A national position paper issued by Novartis Pharma Services Romania estimated that approximately 600,000 cases of HF—either primary or secondary—were documented in Romania [5]. The average LOS for patients with chronic heart failure (CHF) was 6.7 days, with one in five patients experiencing at least one HF-related hospital readmission within the subsequent year.

Hypoalbuminemia is commonly observed in CHF and is associated with raised inflammation, oxidative stress, hypervolemia, and edema (including myocardial, pulmonary, and enteral). Additionally, it contributes to diuretic resistance and myocardial electrical instability, affecting approximately 20–25% of patients with CHF [6,7]. A retrospective study of 2280 patients with acute heart failure (AHF) in the intensive care unit demonstrated a significant correlation between hypoalbuminemia and prolonged hospitalization [8]. This finding is further supported by Pokhrel et al., who reported an association between hypoalbuminemia and increased LOS in AHF patients [9].

A meta-analysis of ten studies and a 12.8-month follow-up study identified the neutrophil-to-lymphocyte ratio (NLR) as a significant predictor of mortality in heart failure (HF) patients [10,11]. Delcea et al. further demonstrated that NLR correlates significantly with the New York Heart Association (NYHA) functional class, N-terminal pro-B-type natriuretic peptide (NT-proBNP) levels, left ventricular ejection fraction (LVEF), and the number of comorbidities [12]. Moreover, a previous study revealed a weak but positive association between NLR and LOS in a HF cohort, with the strongest correlation observed in patients with heart failure with mildly reduced ejection fraction [13].

Hu et al. established the neutrophil percentage-to-albumin ratio (NPAR) as an independent predictor of LOS in patients with HF [14]. Furthermore, elevated NPAR values have been independently associated with increased all-cause mortality at 90 days as well as at one and two years in the HF population [15,16].

Red blood cell distribution width (RDW) quantifies the degree of anisocytosis and has been associated with adverse clinical outcomes including the development of CHF [17]. In obese individuals, RDW does not correlate with inflammatory biomarkers [18]. Jing et al. demonstrated significantly higher RDW levels in chronic obstructive pulmonary disease (COPD) patients who also had pulmonary embolism, further showing an association between RDW, COPD severity, and the survival rates [19,20]. In the context of HF, combining RDW with NT-proBNP enhances prognostic accuracy across all clinical subtypes, particularly in diabetic and hypertensive patients [21]. Moreover, RDW serves as a robust predictor of both mortality and rehospitalization in HF patients with chronic kidney disease (CKD) [22]. A study investigating RDW thresholds in this population identified an RDW ≥ 15.5% as a predictor of in-hospital mortality in HF patients with CKD [23].

Fibrinogen can increase up to tenfold in response to acute-phase inflammatory reactions, thereby playing a central role in both thrombogenesis and systemic inflammation [24].

Elevated RDW-to-albumin ratio (RAR) has been associated with increased short-term (30-day) and long-term (1-year) mortality in patients with HF [25]. Extensive research has established a link between higher RAR levels and worse renal outcomes in patients with CKD [26]. RAR has also been implicated in various disease pathways, including its association with diabetes mellitus severity and complications, as a prognostic predictor in acute myocardial infarction, or as an independent predictor of in-hospital mortality in critically ill COPD patients [27,28,29,30,31].

In a retrospective study, Xu et al. found that the fibrinogen-to-albumin ratio (FAR) independently predicted short- and long-term HF outcomes including 90-day and 1-year mortality and LOS [32]. Similarly, Yang et al. reported that elevated FAR levels predicted mortality in CHF, regardless of HF phenotype [33].

The 2023 focused update of the 2021 ESC Guidelines for the Diagnosis and Treatment of Acute and Chronic Heart Failure includes NT-proBNP as a key biomarker in the diagnostic evaluation of HF [34]. While both B-type natriuretic peptide (BNP) and NT-proBNP serve as diagnostic markers for HF, NT-proBNP’s longer plasma half-life renders it a more stable indicator of cardiac dysfunction [35,36,37]. Welsh et al. proposed a refined approach to the conventional 125 pg/mL cut-off values, emphasizing that NT-proBNP interpretation should incorporate age- and sex-specific thresholds to improve diagnostic accuracy [38].

This pilot study was designed to evaluate the prognostic utility of three novel biomarker ratios—the NT-proBNP-to-albumin ratio (NTAR), the RDW-to-fibrinogen ratio (RFR), and the RDW-to-estimated glomerular filtration rate ratio (RGR)—in predicting the hospital length of stay (LOS), extended LOS (ELOS), in-hospital mortality, and 6-month all-cause mortality. The study’s innovation lies in introducing these previously unexplored laboratory-derived indices to address an existing gap in predicting hospitalization duration and clinical outcomes. To validate our findings, the performance of these novel biomarker ratios were compared with six established biomarkers (albumin, NLR, NPAR, RAR, FAR, and NT-proBNP) alongside key clinical parameters such as age, gender, NYHA functional class, and comorbidity burden. Multivariate analyses were employed to develop and refine predictive models for mortality, thereby delineating the most influential prognostic factors.

## 2. Materials and Methods

This observational, retrospective pilot study was conducted in the Department of Internal Medicine II—Cardiology at the County Emergency Clinical Hospital Targu Mures, Romania, spanning from 1 January 2022 to 31 July 2024. As the first pilot study of its kind in Romania, Eastern Europe, and abroad, it investigated novel HF biomarker assays by correlating exploratory ratios (NTAR, RFR, RGR) with clinical outcomes such as LOS and mortality, benchmarking them against established markers. Multivariate analyses were employed to quantify the incremental prognostic value of these biomarkers, accurately estimating the effect sizes to inform and power subsequent larger-scale trials.

The principal dataset comprised 615 hospital admissions extracted from the electronic database using ICD-10 (version 2019) discharge codes I50, I50.0, I50.1, and I50.9.

Inclusion criteria comprised patients with CHF who experienced either sudden or gradual clinical deterioration, classified as NYHA functional classes II–IV and a minimum LOS of at least 48 h [39].

Exclusion criteria encompassed active inflammation, confirmed acute infections, sepsis, malignancies, autoimmune disorders, primary or infectious hepatic diseases, primary hematologic malignancies, myelodysplastic syndromes, or CKD stage V (regardless of dialysis status). Patients with incomplete datasets or those discharged at their own request were also excluded from the analysis. After applying these criteria, the final cohort consisted of 382 admissions.

Demographic and clinical variables collected in this study included age, gender, geographical background (urban or rural), body mass index (BMI), NYHA functional classification, comorbidities—presence of coronary artery disease (CAD), history of myocardial infarction, systemic hypertension, valvular heart disease (≥moderate severity), prior valvular surgery, atrial fibrillation (AF), history of myocarditis, type II diabetes mellitus (T2DM), COPD, anemia, CKD, and dysthyroidism.

Excess body weight was defined as a BMI ≥ 25 kg/m^2^.

Atrial fibrillation was classified separately, irrespective of its presentation (paroxysmal, persistent, or permanent) or concurrent atrial flutter. Anemia was defined using sex-specific hemoglobin thresholds, with values < 12 g/dL in women and <13 g/dL in men [40].

In accordance with the ESC guidelines in place at the time of the study, HF phenotypes were categorized based on LVEF: (i) HF with reduced ejection fraction (HFrEF); (ii) HF with mildly reduced ejection fraction (HFmrEF); and (iii) HF with preserved ejection fraction (HFpEF) [34,41].

ELOS was designated as any hospital stay exceeding 7 days.

Fasting blood samples were collected within the first two hours of admission from the cephalic, basilic, or median cubital veins. All analyses were performed in an ISO-15189-certified laboratory using the following systems: (i) Sysmex XN-550™ (Sysmex Corporation, Kobe, Japan) for complete blood count; (ii) Konelab Prime 60i (Thermo Fisher Scientific Inc., Waltham, MA, USA) for creatinine, erythrocyte sedimentation rate (ESR), and fibrinogen; and (iii) Nano-Checker™ 710 Reader (Nano-Ditech Corporation, Cranbury, NJ, USA) for NT-proBNP quantification.

Laboratory parameters analyzed included complete blood count (including absolute and percentage neutrophil count, absolute lymphocyte count, RDW), biochemical indices (albumin, creatinine, estimated glomerular filtration rate—eGFR), and inflammatory biomarkers ESR, fibrinogen as well as NT-proBNP.

In-hospital heart failure HF management was in-line with the established guidelines, and patients demonstrated clinical improvement at discharge, evidenced by symptomatic relief and/or an improvement in NYHA functional class. The 6-month all-cause mortality was assessed in compliance with GDPR regulations via structured telephone follow-ups, identifying 29 deaths (7.88%) among the discharged patients.

### 2.1. Proposed New Biomarkers

All newly proposed biomarkers were defined as the ratio between the absolute values of established biomarkers, irrespective of their measurement units. This approach allows for a dimensionless assessment, enhancing the comparability and clinical applicability of these novel indices. By eliminating the influence of measurement units, these new biomarkers may offer a standardized method for evaluating disease progression, treatment response, and patient prognosis across diverse populations and laboratory settings.

### 2.2. RDW-to-Fibrinogen Ratio (RFR)

A composite biomarker proposed for evaluation is the red cell distribution width-to-fibrinogen ratio (RFR), which underscores the interplay between the interconnection of anemia-related oxygen delivery impairment and systemic inflammation. In HF, an elevated RFR may reflect the complex synergy between inflammation burden and vascular dysfunction. Consequently, a higher RFR could signal increased inflammatory and hemodynamic stress, potentially serving as a predictor of prolonged hospitalization and elevated mortality risk in HF patients. RFR was calculated by dividing the absolute value from the standard deviation of RDW to absolute values of fibrinogen: $RFR=\frac{RDW\;(fl)}{Fibrinogen\;(\frac{g}{L})}$.

### 2.3. RDW-SD-to-eGFR Ratio (RGR)

The kidney exerts an indirect but significant role in modulating RDW. Renal dysfunction impairs erythropoietin production, contributing to anemia and creating a heterogeneous red blood cell population, thereby increasing RDW. In clinical practice, the eGFR is widely recognized as a robust marker of renal function. In HF, reduced cardiac output diminishes renal perfusion, leading to a consequent decline in eGFR. The RDW-to-eGFR ratio (RGR) was calculated by dividing the RDW standard deviation (expressed in femtoliter-fl) by eGFR (mL/min/1.73 m^2^), determined by applying the 2021 CKD-EPI creatinine formula: $RGR=\frac{RDW(fl)}{eGFR\;(mL/min/1.73m²)}$.

### 2.4. NT-proBNP-to-Albumin Ratio (NTAR)

The NT-proBNP-to-albumin ratio (NTAR), as a novel biomarker, encapsulates myocardial wall stress and systemic congestion in HF. The rationale behind this biomarker is based on the pathophysiological interplay between cardiac overload and hepatic congestion. Elevated cardiac stress stimulates the release of natriuretic peptides, specifically NT-proBNP, while advanced stages of HF contribute to hepatic congestion, disrupting cellular homeostasis and impairing albumin synthesis. NTAR was computed as the base-10 logarithm of the NT-proBNP-to-albumin ratio: $NTAR=Log(10)\frac{NT-proBNP\;(\frac{pg}{mL})}{Albumin\;(\frac{mg}{dL})}$.

### 2.5. Statistical Analysis

All data (382 cases) were compiled using Microsoft**^®^**Excel**^®^**2016 MSO version 16.0.4738.1000 for Windows (Microsoft Corporation, Redmond, WA, USA) and subsequently analyzed using MedCalc^®^ Statistical Software version 23.1.6 (MedCalc Software Ltd., Ostend, Belgium; https://www.medcalc.org; accessed on 12 February 2025). Statistical significance was considered when *p* ≤ 0.05.

Categorical data are presented as frequencies (percentages), while continuous data are expressed as the median with interquartile range (IQR). Normality was assessed using the Kolmogorov–Smirnov test, which indicated a non-Gaussian distribution for all continuous variables. Consequently, Spearman’s rank correlation was applied for non-parametric data analysis.

The NT-proBNP values exhibited substantial variability, prompting logarithmic transformation (base-10). Outliers (*n* = 57) were identified via the robust regression and outlier removal (ROUT) test. The retention of outliers was justified to preserve biological relevance.

### 2.6. Logistic Regression Analysis

Logistic regression was employed to categorize data into dichotomous variables (0/1). The odds ratio (OR) was calculated with the 95% confidence interval (CI) to quantify the associations between the predictors and outcomes, and model validity was assessed using the Hosmer–Lemeshow goodness-of-fit test (*p* > 0.05 indicating adequate fit).

### 2.7. ROC Curve Analysis

To evaluate predictive accuracy, receiver operating characteristic (ROC) curve analysis was performed, and the area under the curve (AUC) was calculated with 95% CI. Comparisons across ROC curves were performed via DeLong’s test. Cut-off values that best balanced sensitivity and specificity were identified using the Youden index [42].

### 2.8. Cox Proportional Hazards Model

To minimize overfitting, the Cox proportional hazards model employs a stepwise selection method to examine the relationships between clinical variables, multiple biomarkers, and in-hospital mortality. The model’s performance was assessed using Harrell’s C-index with 95% CI, and the findings were interpreted with reference to the previous literature to maintain methodological rigor [43].

## 3. Results

### 3.1. Cohort Characteristics

A total of 382 hospital admissions for CHF were analyzed. The median age of the study population was 71 years (IQR: 61–77 years) with males comprising 54.97% of the studied cases. Approximately two-thirds of the cohort resided in urban areas. HFrEF was the most prevalent phenotype, and half of the patients presented with NYHA functional class III. Table 1 summarizes the main characteristics of the cohort.

### 3.2. Comorbidities

On average, half of the patients had a minimum of five comorbidities from the thirteen comorbidities assessed. There was no statistically significant difference for comorbidities regarding geographical background (*p* = 0.592), NYHA functional class assessment (*p* = 0.198), HF phenotype (*p* = 0.85), or hospitalization duration (LOS ≤ 7 days or ELOS; *p* = 0.102).

In our cohort, 74.08% of patients exhibited excess body weight, with a median BMI of 28.73 kg/m^2^; however, no significant differences were observed across the HF phenotypes. Moderate to severe valvular heart disease was among the most common comorbidities, closely followed by hypertension. Ischemic heart disease was present in approximately 44% of cases, with 28% of subjects having a documented history of myocardial infarction. T2DM was identified in about one-third of patients, while COPD affected roughly one-sixth. Anemia was detected in 27.48% of individuals, with a higher prevalence among women (56.19% of the anemic subgroup). Additionally, CKD was present in 31.15% of patients, and thyroid dysfunction was reported in approximately 20% of the cohort, with similar rates observed in both the HFrEF and HFpEF populations.

### 3.3. Length of Hospital Stay

Median LOS for the entire cohort was 7 days with no significant difference among the three HF phenotypes. A total of 43.19% of patients required ELOS. Patients requiring ELOS were significantly more likely to present with a higher NYHA functional class compared with patients with shorter admissions (≤7 days; *p* = 0.001).

### 3.4. Laboratory Data

Eight biomarker ratios, calculated from hematological indices, were assessed for the entire study population (Table 2). A significant association was found between RGR values and CKD stages (*p* < 0.001), with higher RGR levels corresponding to more advanced renal disease stages.

### 3.5. Biomarkers and LOS

Regarding LOS, significant positive correlations were observed with NTAR, RAR, and Log(10) NT-proBNP, whereas albumin showed a significant negative correlation. These findings suggest a potential interplay between these biomarkers and the overall hospital stay, as detailed in Table 3.

In patients with an LOS ≤ 7 days, low but significant positive associations were observed with Log(10) NT-proBNP, NTAR, RGR, RAR, FAR, and NPAR, while albumin exhibited a low but significant negative correlation.

In contrast, for patients with ELOS, RAR exhibited a moderate positive correlation. Additionally, low positive correlations were observed for NTAR, RFR, Log(10) NT-proBNP, and NLR. Albumin maintained a low negative correlation with ELOS.

### 3.6. Biomarkers and ELOS Analysis

To identify patients at risk of ELOS within the entire cohort, logistic regression analysis was conducted. The analysis identified that RAR, NTAR, albumin, and Log(10) NT-proBNP were the most reliable predictors of ELOS in HF patients, based on both the AUC values and model validation (Table 4). Among these, RAR exhibited the highest AUC, though the differences among the biomarkers were marginal.

These biomarkers demonstrated sufficient to good discriminatory power (AUC: 0.690–0.700), highlighting their potential clinical utility in assessing the risk of prolonged hospitalization.

Conversely, FAR, NLR, RGR, NPAR, and RFR exhibited poor discriminatory ability (AUC < 0.6), limiting their clinical applicability in assessing the risk of prolonged hospitalization.

Notably, the Hosmer–Lemeshow test confirmed the validity of the predictive models, particularly those with high discriminatory power derived from RAR, albumin, Log(10) NT-proBNP, and NTAR. These findings underscore the significance of inflammatory markers, myocardial stress indicators, nutritional status, and anemia in predicting prolonged hospitalization in HF patients.

**Figure 1:**
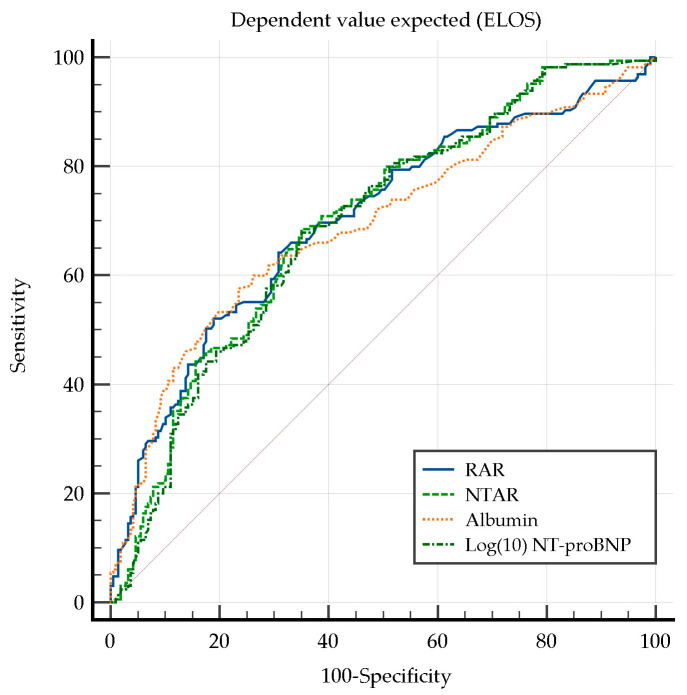
Comparison of the ROC curves for ELOS. NTAR, NT-proBNP-to-albumin ratio; RAR, red cell distribution width-to-albumin ratio.

ROC analysis was performed to compare the predictive performance of the biomarkers with AUC > 0.6—RAR, NTAR, albumin, and Log(10) NT-proBNP in identifying the optimal model for ELOS (Figure 1).

NTAR demonstrated a superior predictive performance compared with Log(10) NT-proBNP (difference between AUC = 0.007, 95% CI: 0.003–0.011, *p* = 0.005), suggesting that albumin enhances the prognostic value of NT-proBNP. However, the AUC of NTAR did not differ significantly from those of RAR and albumin, suggesting comparable discriminatory power among these biomarkers in predicting prolonged hospitalization.

### 3.7. Biomarkers and In-Hospital Mortality

In-hospital mortality was recorded in 14 patients (3.6%), with a male-to-female death ratio of 1.8:1. The overall mortality ratio between patients from urban and rural areas was 1.15:1. Notably, 93% of in-hospital deaths occurred in patients classified as NYHA functional class III or IV.

Univariate logistic regression identified multiple significant predictors of in-hospital mortality (Table 5).

RGR exhibited the highest predictive power for in-hospital mortality (AUC = 0.785) and a strong association with mortality risk (OR = 3.591), underscoring the impact of renal dysfunction and erythropoietic alterations in HF prognosis.

Similarly, NTAR, Log(10) NT-proBNP, RAR, and albumin demonstrated strong predictive capacities, highlighting the interplay of inflammation, cardiac dysfunction, systemic congestion, and anemia. NLR was also significantly associated with mortality, suggesting a substantial role of systemic inflammation, with moderate discriminative power. In contrast, albumin emerged as a protective factor (OR = 0.275), reinforcing its prognostic value in HF patients.

The Hosmer–Lemeshow test confirmed good model fitting, supporting the robustness and reliability of these mortality predictors.

In this analysis, NPAR, FAR, and RFR did not yield significant ROC curves comparison analysis.

**Figure 2:**
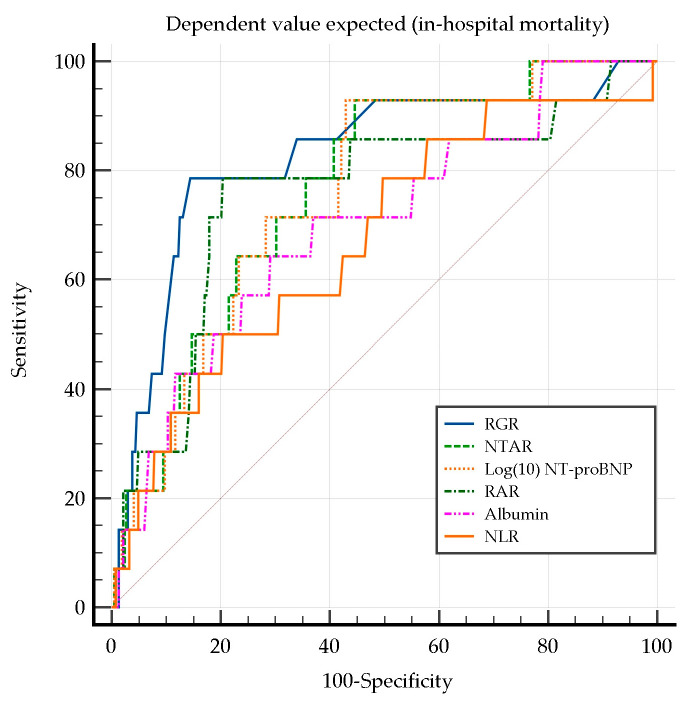
Comparison of ROC curves for in-hospital mortality. NLR, neutrophil-to-lymphocyte ratio; NTAR, NT-proBNP-to-albumin ratio; RAR, red cell distribution width-to-albumin ratio; RGR, red cell distribution width-to-eGFR ratio.

Furthermore, the ROC curve comparison did not reveal any significant pairwise differences among the well-fitted biomarker models in predicting in-hospital mortality (*p* < 0.05) (Figure 2).

Cox proportional hazards analysis using stepwise selection identified RGR as the sole independent predictor of in-hospital mortality among the nine studied biomarkers (HR: 2.18, 95% CI: 1.264–3.758, *p* = 0.005). The overall model demonstrated statistical significance (*p* = 0.015) and exhibited good predictive ability as indicated by Harrell’s C-index = 0.700 (95% CI: 0.470–0.930).

An additional analytical approach involved developing two models to evaluate the relationship between hospitalization duration and multiple clinical factors, using discharge status—alive—as the censoring criterion:

- Model 1 included albumin and the eight biomarker ratios;
- Model 2 incorporated the same eight biomarkers, alongside age, gender, NYHA functional class, and the total number of comorbidities.

Cox logistic regression analysis performed using the stepwise method identified RAR and NTAR in model 1 as significant predictors associated with a reduced likelihood of being discharged alive (Table 6). In model 2, when the NYHA functional class was incorporated, it emerged as an additional significant predictor of hospital mortality alongside RAR and NTAR.

Both models were validated through overall model fitting (*p* < 0.001) and demonstrated good concordance, as confirmed by Harrell’s C-index, reinforcing their predictive accuracy beyond chance.

For model 2, the cumulative hazard analysis revealed that by day 10, the probability of remaining hospitalized while alive declined to approximately 27.1%, whereas by day 20, only ~1.9% of patients remained in the hospital without being discharged or deceased.

### 3.8. Biomarkers and 6-Month All-Cause Mortality

Approximately 69% of deceased patients within six-months after being discharged were admitted in advanced clinical severity of functional class (NYHA III or IV), mirroring the pattern observed in in-hospital mortality, where higher NYHA functional classes were similarly linked to increased death rates.

Univariate analysis identified NTAR, Log(10) NT-proBNP, RGR, NLR, and FAR as the strongest predictors of 6-month all-cause mortality, emphasizing the roles of systemic congestion, cardiac stress, inflammation, and nutritional imbalances in long-term outcomes. These findings extend previous observations regarding in-hospital mortality. Predictors of 6-month all-cause mortality using univariate logistic regression analysis are presented in Table 7.

Although albumin remained a relevant prognostic marker post-discharge, its discriminative power for long-term mortality was limited. The Hosmer–Lemeshow test confirmed robust model fitting, especially for key inflammatory and cardiac biomarkers.

**Figure 3:**
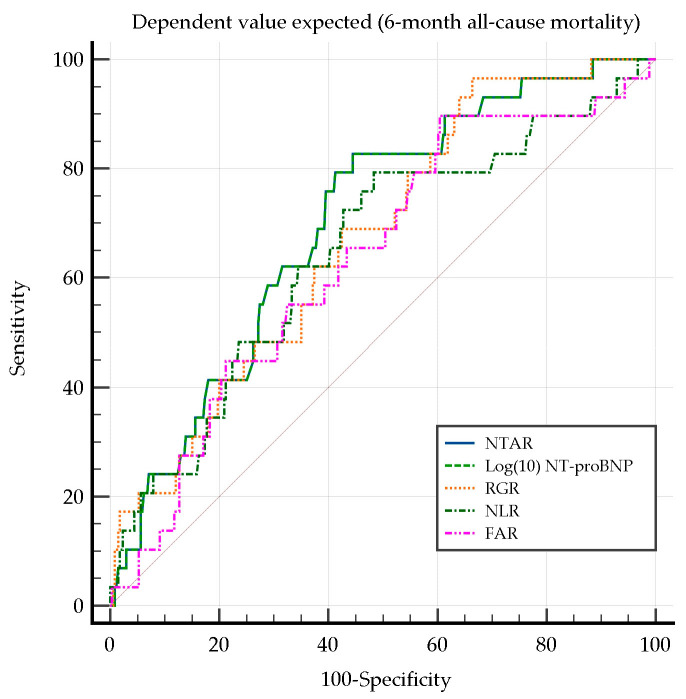
Comparison of ROC curves for 6-month all-cause mortality. FAR, fibrinogen-to-albumin ratio; NLR, neutrophil-to-lymphocyte ratio; NTAR, NT-proBNP-to-albumin ratio; RGR, red cell distribution width-to-eGFR ratio.

The ROC curve comparison did not reveal any significant differences in predicting the 6-month all-cause mortality (Figure 3). Despite the NPAR and RAR models’ acceptable discriminatory power (AUC) and satisfactory model fitting (Hosmer–Lemeshow test), their contributions within the regression framework were neither sufficiently strong nor precise to establish a statistically significant effect (OR).

A summary of the study findings is presented in Table 8, offering a comprehensive overview of the key significant parameters and predictive models identified throughout the analysis. This structured format enhances the interpretation of the study’s main outcomes, facilitating a clearer understanding of the most relevant prognostic factors.

## 4. Discussion

This pilot study explored the prognostic value of both the novel proposed and well-established biomarkers in relation to LOS, ELOS, in-hospital mortality, and 6-month all-cause mortality in a cohort of 382 hospitalized HF patients. Our findings underscore the necessity of incorporating inflammatory, metabolic, hematologic, and renal biomarkers into predictive models, as individual parameters may not fully capture the complexity of HF prognosis.

To our knowledge, this is the first study to propose and evaluate RFR, RGR, and NTAR as potential prognostic markers in HF-related hospitalization and mortality. Given the increasing recognition of inflammatory and metabolic markers in HF progression, our results suggest that these novel ratios may offer additional clinical insights beyond traditional biomarkers such as NT-proBNP and albumin.

### 4.1. Prior Biomarkers and Identified Cut-Offs

Albumin demonstrated a negative correlation with LOS (r = −0.36, *p* < 0.001), with levels above 3.9 g/dL exerting a protective effect against prolonged hospitalization. In contrast, albumin cut-off of <3.79 g/dL was indicative of increased risk for in-hospital mortality. Our findings align with existing medical literature that associates hypoalbuminemia with ELOS in patients with AHF [8,9,44,45]. This relationship suggests that albumin supplementation during hospitalization may reduce hospital stay. However, a study investigating albumin administration in HF patients reported paradoxical findings, with albumin therapy being linked to prolonged intensive care unit stays, ELOS, and increased in-hospital mortality [46].

As a widely studied biomarker, NLR demonstrated a limited correlation with hospitalization duration and was not validated as a predictor for prolonged hospitalization (r = 0.19, *p* < 0.001). However, its prognostic significance in mortality was evident: a NLR cut-off > 4.68 was associated with a 14.2% increase in in-hospital mortality, while a threshold > 2.82 predicted a 15.1% increase in 6-month all-cause mortality. Durmus et al. identified an NLR cut-off of 5.1 for predicting all-cause mortality after a mean follow-up of 12.8 months [10]. Our findings suggest that cut-off values tend to decrease when predicting long-term prognosis, likely reflecting the cumulative impact of chronic inflammation and comorbidities, which progressively worsen outcomes. Similarly, Wu et al. analyzed NLR across four quartiles and confirmed its independent predictive value for all-cause mortality in HF. However, they observed a decline in its predictive performance over time [47]. Köse et al. reported that stable CHF patients with NLR > 3.7 had a 3.4-fold increased risk of one-year mortality, while Che et al. found that an NLR > 4 in critically ill HF patients correlated with increased 30-day mortality [48,49]. Our findings align with prior research, reaffirming NLR as a valuable prognostic marker in HF, particularly in predicting mortality risk across different time frames.

NPAR exhibited no significant correlation with hospitalization duration (r = 0.12, *p* = 0.150). However, in the logistic regression model, it was significantly associated with in-hospital mortality (OR: 1.054, *p* < 0.001), with the model demonstrating adequate model fitting. Despite this, its discriminatory ability between survivors and non-survivors was limited, as indicated by a modest AUC of 0.623, which was not statistically significant (*p* = 0.125). These findings suggest that while NPAR may contribute to mortality risk assessment, it lacks the robustness to function as a standalone predictive marker and should be interpreted alongside other clinical parameters. NPAR was also independently associated with 6-month all-cause mortality; however, its discriminative power remained suboptimal. Further studies incorporating larger sample sizes and additional predictive factors may enhance the model’s performance. Previous research has identified NPAR as a weak independent predictor of increased long-term all-cause mortality in HF patients, underscoring the need for further validation before its routine clinical application [47]. Furthermore, NPAR outperformed neutrophil percentage and albumin in predicting 90-day, one-year, and two-year all-cause mortality in HF patients [15]. A similar association was observed by Kurkiewicz et al. in patients with advanced HF [17]. Our findings contrast with those of Hu et al., who reported a correlation between NPAR and LOS. However, their analysis categorized NPAR into three subgroups, whereas we assessed the entire cohort using logistic regression to identify patients at risk for prolonged hospitalization. Additionally, Hu et al. found a moderate positive Pearson correlation (r = 0.325) between NPAR and LOS, further supporting the potential role of inflammatory and nutritional markers in HF prognosis [14].

RAR exhibited a moderate positive correlation with LOS (r = 0.42, *p* < 0.001), highlighting its potential role in anemia, inflammation, nutritional status, and congestion in HF. A cut-off value > 3.55 was associated with a 2.37-fold increased risk of ELOS, while values exceeding 4.16 were significantly linked to higher in-hospital mortality. Multivariate Cox analysis further confirmed RAR’s relevance in influencing survival odds. Although RAR demonstrated a weak but statistically significant discriminative ability between survivors and non-survivors (AUC = 0.636, *p* = 0.005), its independent association with in-hospital mortality was not statistically significant. The model exhibited good fitting, suggesting that while RAR contributes to mortality risk stratification, it may not serve as an independent predictor in this context. Prior research supports its potential prognostic value in both short- and long-term HF outcomes, warranting further investigation into its clinical utility [25].

Although FAR was modestly correlated with LOS (r = 0.19, *p* = 0.001), it lacked predictive discrimination power to identify prolonged hospitalization and in-hospital mortality. However, a cut-off value > 8.79 at admission was linked to an increased risk of 6-month all-cause mortality. Xu et al. previously reported an association between FAR and LOS as well as 90-day and one-year mortality in HF patients [32]. Similarly, Yang et al. corroborated FAR’s prognostic significance, identifying a 6-month mortality cut-off of 9.06, a value closely aligned with the threshold established in this study [33].

Log(10) NT-proBNP exhibited a positive correlation with LOS (r = 0.40, *p* < 0.001), with higher levels (>3.21, approximately 1622 pg/mL) significantly increasing the likelihood of ELOS (OR: 2.41). For mortality prediction, Log(10) NT-proBNP thresholds >3.32 and >2.62 were associated with an elevated risk of in-hospital and 6-month all-cause mortality, respectively. These findings are consistent with the existing literature reinforcing NT-proBNP as a robust predictor of both acute and chronic HF, capable of forecasting both short- and long-term mortality [50,51,52]. Notably, the prognostic value of NT-proBNP remains independent of patients’ body mass index [53]. Interpreting Log(10) NT-proBNP in clinical practice suggests that each tenfold increase in NT-proBNP level corresponds to prolonged LOS and an increased risk of extended hospitalization, in-hospital mortality, and 6-month all-cause mortality.

### 4.2. Proposed Biomarkers and Identified Cut-Offs

Although RFR did not correlate with length of stay (LOS) (r = 0.06, *p* < 0.001), it was independently associated with an increased risk of both prolonged hospitalization and in-hospital mortality, with the model demonstrating good fit. However, its discriminatory ability to differentiate between LOS ≤ 7 days and ELOS as well as between the survivors and non-survivors remained poor at both time points. These findings suggest that while the interplay between anemia and inflammation may influence patient outcomes, its overall impact appears to be limited. Consequently, the prognostic value of RFR appears minimal—potentially due to the limited contribution of fibrinogen—and future studies should aim to clarify the individual influence of its components.

In our study, RGR emerged as a novel biomarker with significant prognostic value. Although it exhibited a weak positive correlation with LOS (r = 0.20, *p* < 0.001) and performed poorly in identifying patients at risk for prolonged hospitalization, its predictive strength for mortality was notable. At admission, an RGR value > 1.23 was strongly associated with increased in-hospital mortality (OR: 1.739), while an RGR > 0.49 correlated with a higher risk of 6-month all-cause mortality (OR: 2.562). Among the nine biomarkers analyzed, RGR was the only significant predictor of in-hospital mortality in the Cox univariate analysis (HR: 2.18, 95% CI: 1.264–3.758, *p* = 0.005) with overall good model significance (*p* = 0.015) and exhibited overall good model fitting (Harrell’s C-index = 0.700, 95% CI: 0.470–0.930). Biologically, elevated RGR may reflect increased erythrocyte width variability coupled with reduced eGFR, highlighting the complex interplay between the cardiorenal axis and renal-anemia metabolism. Patients with high RGR levels are more susceptible to rapid clinical deterioration and have a lower likelihood of survival to discharge. These findings align with previous research linking red cell distribution width variations to HF mortality, further supporting the role of erythropoietic and renal dysfunction in HF prognosis [21,23]. Furthermore, previous studies have identified that even a slight decline in eGFR serves as a significant predictor of increased mortality in HF [54]. The renal dysfunction observed in CKD establishes a bidirectional interaction with HF, exacerbating disease progression, limiting therapeutic options, and contributing to recurrent hospitalizations and increased mortality [55,56].

Across the 382 studied cases, we observed a moderate positive correlation between NTAR and LOS (r = 0.45, *p* < 0.001). NTAR provided additional prognostic insight beyond NT-proBNP alone. A cut-off > 2.7 was associated with a 4.46-fold increase in in-hospital mortality and a 3.2-fold increase in 6-month all-cause mortality. Since NTAR is calculated as Log(10) NT-proBNP/albumin, its fluctuations are primarily driven by NT-proBNP levels, while albumin exerts a modulating effect. Our findings suggest that elevated NTAR serves as a dual predictor of both prolonged hospitalization and increased mortality risk. Moreover, NTAR outperformed Log(10) NT-proBNP in the predictive models, reinforcing the clinical advantage of integrating these biomarkers for risk assessment. Previous studies indicate that malnutrition, as reflected by hypoalbuminemia, exacerbates HF prognosis. Pagnesi et al. highlighted the combined role of NT-proBNP and albumin in predicting one-year all-cause mortality in HF patients [57].

These analyses underscore the complex interplay among renal, cardiac, inflammatory, and metabolic dysfunctions in long-term mortality risk stratification, emphasizing the necessity for a comprehensive biomarker-based approach across different stages of HF management.

### 4.3. Limitations and Future Directions

Although this pilot study’s retrospective, single-center design provides essential preliminary insights, its inherent limitations call for external validation through larger, multicenter investigations. Future research should systematically incorporate detailed medication regimens at admission and throughout hospitalization to assess their impact on LOS, while a more comprehensive evaluation of the comorbidities and HF-related symptoms may enhance the precision of predictive models.

Our findings underscore the importance of bridging the gap between our novel biomarkers—NTAR, RGR, and RFR—and their practical clinical applications. NTAR, serving as a composite marker of cardiac stress and nutritional status, not only differentiates stable CHF patients from those at higher risk for prolonged hospitalization and mortality, but also demonstrates a superior predictive value over NT-proBNP alone. Meanwhile, RGR appears particularly valuable in CHF patients with concomitant renal dysfunction, guiding nephrology referrals and renal-protective interventions, whereas RFR—though less pronounced—could serve as a secondary marker in patients with chronic inflammation or anemia, warranting further investigation.

For therapeutic decision-making, integrating NTAR and RGR into clinical algorithms could enable more tailored treatment strategies, for example, prompting aggressive decongestive therapy and nutritional support in patients with elevated NTAR, and facilitating individualized diuretic regimens with closer renal monitoring in those with high RGR. We advocate for the inclusion of these biomarkers in existing CHF risk models alongside NT-proBNP, eGFR, and inflammatory markers, and for broadening the analytical scope to encompass a wider range of comorbidities and HF-related phenotypes to deepen our understanding of disease progression.

Furthermore, novel biomarkers such as S100A8/A9—previously investigated in post-acute myocardial infarction HF—may further enhance risk stratification when combined with the established risk factors [58,59]. Considering that myocardial infarction is a major contributor to HF and that targeted therapies addressing innate immunity remain lacking, future research should prioritize interventions aimed at modulating inflammation as a key driver of HF progression. Such efforts hold significant promise for improving both short- and long-term clinical outcomes in HF populations [60].

## 5. Conclusions

This pilot study highlights the prognostic importance of integrating both conventional and novel biomarkers to predict LOS, ELOS, and mortality in CHF. Notably, NTAR and RGR outperformed traditional markers including NT-proBNP alone. These findings underscore the multifactorial nature of CHF progression, shaped by inflammation, metabolic dysfunction, renal impairment, and myocardial stress.

Although NT-proBNP remains central to risk assessment, its predictive accuracy is significantly enhanced when combined with albumin and RDW. Future multicenter investigations are needed for external validation and further refinement of these predictive models. Broadening biomarker-based approaches—particularly those incorporating inflammatory, renal, and metabolic factors—offers a promising pathway for personalized risk stratification and improved clinical decision-making in CHF management.

## Figures and Tables

**Table 1:** Characteristics of the entire cohort.

Parameter	Entire Cohort (*n* = 382)
Age (years, median)	71 (61–77)
Male (*n*, %)	210 (54.97)
Urban areas (*n*, %)	243 (63.61)
BMI (kg/m^2^, median)	28.73 (24.86–33.71)
**Length of hospital stay**	
LOS (days, median)	7 (5–10.75)
ELOS (*n*, %)	165 (43.19)
ELOS (days, median)	11 (9–14)
**NYHA functional class (*n*, %)**	
II	147 (38.48)
III	194 (50.78)
IV	41 (10.73)
**Comorbidities**	
Total number of comorbidities (median)	5 (4–6)
Excess body weight (*n*, %)	283 (74.08)
CAD (*n*, %)	167 (43.71)
Prior documented myocardial infarction (*n*, %)	92 (28.04)
Hypertension (*n*, %)	306 (80.10)
Valvular heart disease ≥ moderate (*n*, %)	311 (81.41)
Prior valvular surgery (*n*, %)	23 (6.02)
AF (including atrial flutter) (*n*, %)	169 (44.24)
History of myocarditis (*n*, %)	10 (2.61)
T2DM (*n*, %)	135 (35.34)
COPD (*n*, %)	58 (15.18)
Anemia (*n*, %)	105 (27.48)
CKD stages (*n*, %)	114 (29.84)
G1	2 (0.52)
G2	30 (7.85)
G3a	41 (10.73)
G3b	32 (8.37)
G4	9 (2.35)
Dysthyroidism (*n*, %)	75 (19.63)
**HF phenotype (*n*, %)**	
HFrEF	154 (40.31)
HFmrEF	90 (23.56)
HFpEF	138 (36.12)
**Mortality (*n*, %)**	
Overall mortality	43 (11.25)
In-hospital mortality	14 (3.6)
Post-discharge mortality	29 (7.88)

AF, atrial fibrillation; BMI, body mass index; bpm, beats per minute; CAD, coronary artery disease; CKD, chronic kidney disease; COPD, chronic obstructive pulmonary disease; ELOS, extended length of hospital stay, HF, heart failure; HFmrEF, heart failure with mildly reduced ejection fraction; HFpEF, heart failure with preserved ejection fraction; HFrEF, heart failure with reduced ejection fraction; LOS, length of hospital stay; *n*, number; NYHA, New York Heart Association; red cell distribution width; T2DM, type 2 diabetes mellitus.

**Table 2:** Laboratory data and biomarker ratios.

Parameter	Entire Cohort (*n* = 382) (Median, IQR)
**Laboratory data**	
Neutrophils (×10^3^/µL)	5.1 (1.66–6.37)
Neutrophil percentage (%)	66.04 (59.96–73.64)
Lymphocytes (×10^3^/µL)	1.66 (1.25–2.14)
RDW (fl)	46.05 (42.65–50.75)
Albumin (g/dL)	4.06 (3.72–4.31)
Creatinine (mg/dL)	1.03 (0.8–1.29)
eGFR (ml/min/1.73 m^2^)	73.39 (54.75–97.03)
ESR (mm/h)	11.5 (6–24)
Fibrinogen (g/L)	3.86 (3.32–4.64)
NT-proBNP (pg/mL)	1582 (340.95–4946.8)
**Previously studied biomarker ratios**	
NLR	2.9 (0.77–19.6)
NPAR	16.32 (14.48–19.08)
RAR	3.48 (3.1–4.06)
FAR	0.98 (0.81–1.17)
Log(10) NT-proBNP	3.2 (2.53–3.69)
**Proposed biomarker ratios**	
RFR	3.63 (3.02–4.29)
RGR	0.64 (0.47–0.89)
NTAR	2.59 (1.92–3.1)

eGFR, estimated glomerular filtration rate; ESR, erythrocyte sedimentation rate; FAR, fibrinogen-to-albumin ratio; NLR, neutrophil-to-lymphocyte ratio; NPAR, neutrophil percentage-to-albumin ratio; NTAR, NT-proBNP-to-albumin ratio; red cell distribution width; RAR, red cell distribution-to-albumin ratio; RDW, red cell distribution width; RFR, red cell distribution width-to-fibrinogen ratio; RGR, red cell distribution width-to-eGFR ratio.

**Table 3:** Correlations between biomarkers and LOS.

Parameters	Length of Hospital Stay (LOS)					
	Entire Cohort (*n* = 382)		LOS ≤ 7 Days (*n* = 217)		ELOS (*n* = 165)	
	r	*p*	r	*p*	r	*p*
Albumin	−0.36	<0.001	−0.14	0.042	−0.21	0.006
**Previously studied biomarker ratios**						
NLR	0.19	<0.001	0.09	0.165	0.13	0.093
NPAR	0.12	0.150	0.17	0.014	0.06	0.422
RAR	0.42	<0.001	0.21	0.002	0.35	<0.001
FAR	0.19	0.001	0.18	0.007	−0.01	0.885
Log(10) NT-proBNP	0.40	<0.001	0.27	<0.001	0.20	0.009
**Proposed biomarker ratios**						
RFR	0.06	0.218	−0.09	0.195	0.23	0.003
RGR	0.20	<0.001	0.23	0.001	0.08	0.278
NTAR	0.45	<0.001	0.27	<0.001	0.25	0.001

FAR, fibrinogen-to-albumin ratio; NLR, neutrophil-to-lymphocyte ratio; NPAR, neutrophil percentage-to-albumin ratio; NTAR, NT-proBNP-to-albumin ratio; RAR, red cell distribution width-to-albumin ratio; RFR, red cell distribution width-to-fibrinogen ratio; RGR, red cell distribution width-to-eGFR ratio.

**Table 4:** Predictors of ELOS using univariate logistic regression analysis.

Independent Variables	Dependent Value Expected (ELOS)					
	AUC (95% CI)	*p*	OR (95% CI)	*p*	Hosmer– Lemeshow Test, *p*	Associated Criterion to Youden Index J
Albumin	0.690 (0.635–0.745)	<0.001	0.213 (0.127–0.344)	<0.001	0.392	≤3.90
**Previously studied biomarker ratios**						
NLR	0.588 (0.528–0.640)	0.003	1.120 (1.043–1.214)	0.003	0.014	>3.75
NPAR	0.541 (0.481–0.601)	0.163	1.004 (0.977–1.030)	0.783	<0.001	>17.95
RAR	0.700 (0.646–0.753)	<0.001	2.370 (1.801–3.212)	<0.001	0.444	>3.55
FAR	0.597 (0.539–0.654)	0.001	1.129 (1.054–1.210)	<0.001	0.966	>9.55
Log(10) NT-proBNP	0.690 (0.637–0.743)	<0.001	2.410 (1.825–3.239)	<0.001	0.120	>3.21
**Proposed biomarker ratios**						
RFR	0.530 (0.471–0.589)	0.308	1.119 (1.026–1.413)	0.025	0.560	>4.41
RGR	0.585 (0.526–0.644)	0.004	1.297 (1.149–1.510)	<0.001	0.167	>2.42
NTAR	0.697 (0.645–0.750)	<0.001	2.438 (1.858–3.255)	<0.001	0.080	>2.61

AUC, area under the curve; CI, confidence interval; FAR, fibrinogen-to-albumin ratio; LOS, length of hospital stay; NLR, neutrophil-to-lymphocyte ratio; NPAR, neutrophil percentage-to-albumin ratio; NTAR, NT-proBNP-to-albumin ratio; OR, odds ratio; RAR, red cell distribution width-to-albumin ratio; RFR, red cell distribution width-to-fibrinogen ratio; RGR, red cell distribution width-to-eGFR ratio.

**Table 5:** Predictors of in-hospital mortality using univariate logistic regression analysis.

Independent Variables	Dependent Value Expected (In-Hospital Mortality)					
	AUC (95% CI)	*p*	OR (95% CI)	*p*	Hosmer– Lemeshow Test, *p*	Associated Criterion to Youden Index J
Albumin	0.700 (0.553–0.848)	0.007	0.275 (0.111–0.678)	0.007	0.790	≤3.79
**Previously studied biomarkers ratios**						
NLR	0.672 (0.522–0.822)	0.028	1.142 (1.004–1.272)	0.022	0.870	>4.68
NPAR	0.623 (0.466–0.780)	0.125	1.054 (0.980–1.130)	<0.001	0.211	>1.62
RAR	0.756 (0.606–0.906)	<0.001	1.739 (1.234–2.449)	<0.001	0.240	>4.16
FAR	0.619 (0.399–0.838)	0.289	1.181 (0.892–1.564)	0.229	0.294	>8.83
Log(10) NT-proBNP	0.760 (0.646–0.874)	<0.001	4.526 (1.684–12.16)	<0.001	0.420	>3.32
**Proposed biomarkers ratios**						
RFR	0.567 (0.516–0.618)	0.443	1.191 (0.861–1647)	<0.001	0.693	>3.58
RGR	0.785 (0.616–0.953)	<0.001	3.591 (1.191–6.744)	<0.001	0.239	>1.23
NTAR	0.768 (0.655–0.881)	<0.001	4.461 (1.723–11.554)	<0.001	0.863	>2.69

AUC, area under the curve; CI, confidence interval; FAR, fibrinogen-to-albumin ratio; NLR, neutrophil-to-lymphocyte ratio; NPAR, neutrophil percentage-to-albumin ratio; NTAR, NT-proBNP-to-albumin ratio; OR, odds ratio; RAR, red cell distribution width-to-albumin ratio; RFR, red cell distribution width-to-fibrinogen ratio; RGR, red cell distribution width-to-eGFR ratio.

**Table 6:** Cox logistic regression analysis adjusted after stepwise method for models 1 and 2 (endpoint: discharged alive).

Parameters	Time: Admission Length			Overall Model Fit	Harrell’s C-Index
	Exp (b)	95% CI	*p*	*p*	95% CI
**Model 1**	-	-	-	<0.001	0.692 (0.660–0.725)
RAR	0.756	0.659–0.867	<0.001	-	
NTAR	0.666	0.577–0.769	<0.001	-	
**Model 2**	-	-	-	<0.001	0.701 (0.668–0.733)
RAR	0.772	0.673–0.885	<0.001		
NTAR	0.731	0.622–0.859	<0.001		
NYHA functional class	0.768	0.635–0.927	0.006		

CI, confidence interval; NTAR, NT-proBNP-to-albumin ratio; NYHA, New York Heart Association; RAR, red cell distribution width-to-albumin ratio.

**Table 7:** Predictors of 6-month all-cause mortality using univariate logistic regression analysis.

Independent Variables	Dependent Value Expected (6-Month All-Cause Mortality)					
	AUC (95% CI)	*p*	OR (95% CI)	*p*	Hosmer– Lemeshow Test, *p*	Associated Criterion to Youden Index J
Albumin	0.547 (0.437–0.701)	0.402	0.683 (0.328–0.1.42)	0.038	0.206	≤3.41
**Previously studied biomarker ratios**						
NLR	0.649 (0.540–0.759)	0.007	1.151 (1.051–1.261)	0.005	0.249	>2.82
NPAR	0.617 (0.505–0.729)	0.004	1.042 (0.989–1.098)	0.112	0.665	>15.55
RAR	0.636 (0.539–0.733)	0.005	1.246 (0.904–1.717)	0.205	0.546	>3.51
FAR	0.635 (0.531–0.739)	0.011	1.159 (1.031–1.302)	<0.001	0.446	>8.79
Log(10) NT-proBNP	0.702 (0.611–0.792)	<0.001	2.693 (1.513–4.794)	<0.001	0.702	>2.62
**Proposed biomarker ratios**						
RFR	0.553 (0.438–0.669)	0.366	0.939 (0.685–1.286)	0.689	0.835	≤3.06
RGR	0.672 (0.577–0.766)	<0.001	2.562 (1.449–4.529)	0.002	0.259	>0.49
NTAR	0.766 (0.692–0.838)	<0.001	4.185 (2.382–7.353)	<0.001	0.671	>2.69

AUC, area under the curve; CI, confidence interval; FAR, fibrinogen-to-albumin ratio; NLR, neutrophil-to-lymphocyte ratio; NPAR, neutrophil percentage-to-albumin ratio; NTAR, NT-proBNP-to-albumin ratio; OR, odds ratio; RAR, red cell distribution width-to-albumin ratio; RFR; red cell distribution width-to-fibrinogen ratio; RGR, red cell distribution width-to-eGFR ratio.

**Table 8:** Comprehensive summary of the major study findings.

Independent Variables	Entire Cohort LOS	Dependent Value Expected						
		ELOS		In-Hospital Mortality		Discharged Alive	6-Month All-Cause Mortality	
Method Applied	Correlations ^(1)^	Logistic Regression ^(2)^		Logistic Regression ^(2)^		Survival Analysis ^(3)^	Logistic Regression ^(2)^	
		OR	Cut-Off	OR	Cut-Off		OR	Cut-Off
Albumin	Yes, (−)	0.213	≤3.9	0.275	≤3.79	-	-	-
**Previously studied biomarker ratios**								
NLR	Yes, (+)	-	-	1.142	>4.68	-	1.151	>2.82
NPAR	-	-	-	1.054	>1.62	-	-	-
RAR	Yes, (+)	2.37	>3.55	1.739	>4.16	Model 1, 2	-	-
FAR	Yes, (+)	-	-	-	-	-	1.159	>8.79
Log(10) NT-proBNP	Yes, (+)	2.41	>3.21	4.526	>3.32	-	2.693	>2.62
**Proposed biomarker ratios**								
RFR	-	-	-	-	-	-	-	-
RGR	Yes, (+)	-	-	3.591	1.23	-	2.562	>0.49
NTAR	Yes, (+)	2.438	>2.61	4.461	>2.69	Model 1, 2	4.185	>2.69

ELOS, extended length of hospital stay; FAR, fibrinogen-to-albumin ratio; LOS, length of hospital stay; NLR, neutrophil-to-lymphocyte ratio; NPAR, neutrophil percentage-to-albumin ratio; NTAR, NT-proBNP-to-albumin ratio; RAR, red cell distribution width-to-albumin ratio; RDW, red cell distribution width; RGR, red cell distribution-to-eGFR ratio; **^(1)^ Yes, (r > 0.1 or < −0.1); (+), positive correlations; (−), negative correlations. ^(2)^ Adequate model fitting (Hosmer–Lemeshow test *p* > 0.05), AUC > 0.6, *p* < 0.05 for OR and AUC. ^(3)^ Model inclusion, overall model fitted *p* < 0.05, Harrell’s C-index < 0.001.**

## Data Availability

The data presented in this study can be obtained from the corresponding author according to the local and national regulations.

## Abbreviations

The following abbreviations are used in this manuscript:

AF	Atrial fibrillation
AHF	Acute heart failure
AUC	Area under curve
BMI	Body mass index
BNP	Brain natriuretic peptide
CAD	Coronary artery disease
CHF	Chronic heart failure
CI	Confidence interval
CKD	Chronic kidney disease
COPD	Chronic obstructive pulmonary disease
CRP	C-reactive protein
eGFR	Estimated glomerular filtration rate
ELOS	Extended length of hospital stay
ESC	European Society of Cardiology
ESR	Erythrocyte sedimentation rate
FAR	Fibrinogen-to-albumin ratio
HF	Heart failure
HFA	Heart Failure Association
HFmrEF	Heart failure with mildly reduced ejection fraction
HFpEF	Heart failure with preserved ejection fraction
HFrEF	Heart failure with reduced ejection fraction
IQR	Interquartile range
LOS	Length of hospital stay
LVEF	Left ventricle ejection fraction
NLR	Neutrophil-to-lymphocyte ratio
NPAR	Neutrophil percentage-to-albumin ratio
NTAR	NT-proBNP-to-albumin ratio
NT-proBNP	N-terminal prohormone of brain natriuretic peptide
NYHA	New York Heart Association
OR	Odds ratio
RAR	Red cell distribution width-to-albumin ratio
RDW	Red cell distribution width
RDW-SD	Red cell distribution width standard deviation
RFR	Red cell distribution width-to-fibrinogen ratio
RGR	Red cell distribution width-to-estimated glomerular filtration rate ratio
SD	Standard deviation
T2DM	Type 2 diabetes mellitus
